# Comparison of the application value of contrast-enhanced ultrasound and contrast-enhanced CT in puncture biopsy of peripheral pulmonary lesions

**DOI:** 10.3389/fonc.2025.1502356

**Published:** 2025-04-30

**Authors:** Gang Wang, Ji-cheng Zhang, Zhi-hua Wang, Bo Gou, Xiao-lin Liu, Gang Liu, Jian Liu

**Affiliations:** ^1^ Department of Ultrasound Medicine, the Shaoyang Central Hospital of Hunan Province, Shaoyang, Hunan, China; ^2^ Department of Ultrasound, Clinical Medical College and the First Affiliated Hospital of Chengdu Medical College, Chengdu, China; ^3^ Department of Ultrasound Medicine, Nanchong Central Hospital, The Affiliated Nanchong Central Hospital of North Sichuan Medical College, Nanchong Hospital of Beijing Anzhen Hospital Capital Medical University, Nanchong, China

**Keywords:** contrast-enhanced ultrasound, contrast enhanced CT, peripheral pulmonary lesions, percutaneous lung biopsy, diagnostic accuracy

## Abstract

**Objective:**

This study assesses the clinical utility of contrast - enhanced ultrasound (CEUS) in comparison to contrast - enhanced computed tomography (CECT) in the context of peripheral lung mass biopsy. The overarching objective is to establish robust clinical benchmarks that can guide evidence - based decision - making in the field of pulmonary interventional procedures.

**Methods:**

A comparison of 420 patients admitted to our hospital from January 2019 to December 2022 who underwent biopsy using two different guidance methods, including 196 cases in the CEUS-guided biopsy group and 224 cases in the CECT-guided biopsy group. The average number of pleural punctures, puncture time, satisfaction with the first puncture specimen, diagnostic accuracy and complication rate were compared between the two guidance methods.

**Results:**

① Compared with the CECT group, the CEUS-guided group required fewer pleural punctures (2.5 vs. 4.1 times) and shorter puncture time (24 minutes vs. 42 minutes) on average, and the difference was statistically significant (P<0.001). ② In terms of complications, the incidence of pneumothorax (3.1% vs. 8%) was lower in the CEUS group, while the incidence of bleeding (1.5% vs. 3.1%) had no significant difference between the two groups ③ When the diameter of the lesion is <3 cm, the specimen satisfaction and diagnostic accuracy of the CEUS group are lower than those of the CECT group (71.0% vs. 88.3%, 64.5% vs. 86.7%). When the diameter of the lesion is (3 ~ 6cm), the specimen satisfaction and diagnostic accuracy of the CEUS group were higher than those of the CECT group (98.6% vs. 89.6%, 95.8% vs. 85.2%), and the above differences were statistically significant; but when the diameter of the lesion was >6cm, there was no significant difference in specimen satisfaction rate and diagnostic accuracy between the two guidance methods.

**Conclusion:**

CEUS is better than CECT in reducing the number of punctures, shortening puncture time and reducing the incidence of pneumothorax, and is especially suitable for the diagnosis of medium-sized lesions. However, for lesions less than 3 cm in diameter, CECT demonstrated higher specimen satisfaction and diagnostic accuracy. This suggests that diagnostic performance can be optimized by selecting appropriate guidance techniques based on lesion size and risk of complications.

## 1 Introduction

Peripheral pulmonary lesions (PPL) are generally defined as lung lesions in direct contact with the pleura and account for approximately one-third of all lung lesions (1, 2). Among this type of mass, peripheral lung cancer has a relatively poor prognosis, which is mainly due to the fact that it is often at a late stage at the time of diagnosis (3–5). However, numerous studies have shown that early screening, especially in high-risk groups, can significantly reduce mortality and prolong patient survival (3–5).With the rapid development of medical imaging technology, especially computed tomography (CT) technology, the detection rate of peripheral lung masses has increased significantly (6). Percutaneous lung biopsy is an important means of diagnosing PPL and is considered a relatively simple, safe and effective method. According to the existing literature, the diagnostic accuracy of percutaneous lung biopsy for pulmonary malignant tumors is high, ranging from 76% to 97% (6–9). This wide range of accuracy may be related to the biopsy technique, tumor location, and the size of the tumor. In recent years, qualitative diagnosis of chest lesions is mainly based on imaging-guided puncture to obtain lesion tissue to clarify the pathological type, complete molecular level detection, and formulate targeted treatment plans. In clinical practice, percutaneous puncture of PPL is often performed with visual sampling under the guidance of CT and ultrasound. Studies have shown that as the diameter of PPL increases, the probability of necrotic tissue within the lesion increases. Research shows that compared with conventional CT, enhanced CT (CECT) can more clearly display the shape and course of blood vessels in the lesion and provide accurate navigation for percutaneous lung puncture. Percutaneous lung puncture guided by enhanced CT not only has higher satisfaction and accuracy of puncture, but alse the incidence of complications is lower (10, 11). Compared with conventional ultrasound guidance, contrast-enhanced ultrasound (CEUS) has advantages in differentiating atelectatic lung tissue from the active and necrotic areas of tumor lesions, and can clearly display the course of large blood vessels in and around the lesions (12–15). This feature makes CEUS a powerful tool to improve the accuracy of needle biopsy and reduce complications such as postoperative bleeding.

This study retrospectively analyzed the clinical data of 420 patients who underwent percutaneous lung biopsy guided by CEUS or CECT, aiming to compare the differences and effectiveness of two different imaging modalities in guiding percutaneous lung biopsy of peripheral lung masses, as well as to elucidate their respective advantages and disadvantages. Through this study, we hope to provide a strong basis for the rational selection of guidance technology in clinical practice to optimize the diagnostic process, improve diagnostic accuracy, and reduce the risk of complications for patients.

## 2 Data and methods

### 2.1 Patient population

**Figure 1:**
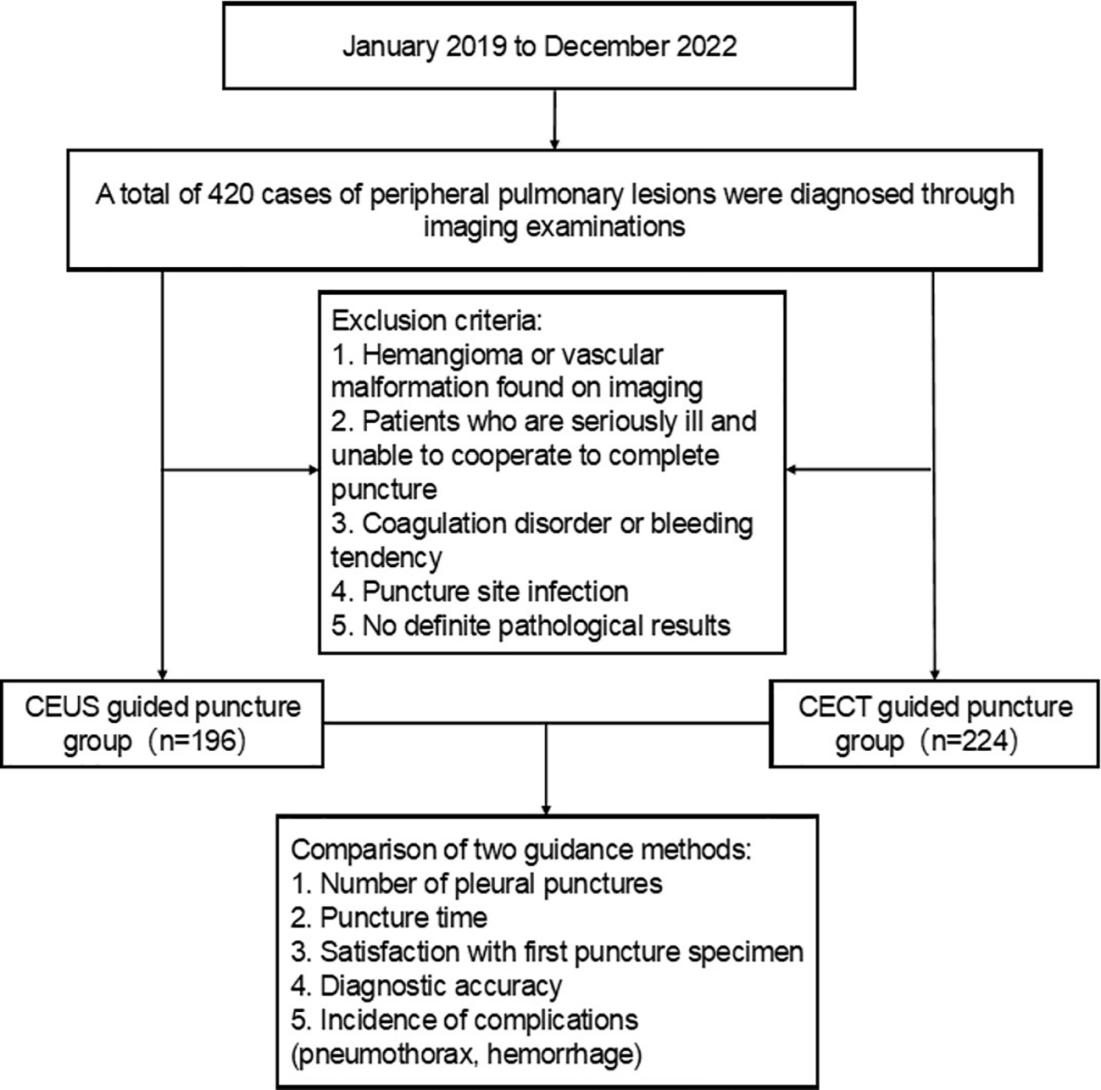
Flow chart for case screening of peripheral pulmonary lesions. CEUS, contrast-enhanced ultrasonography; CECT, contrast-enhanced CT.

The clinical data of patients with peripheral lung masses found in the Department of Respiratory Oncology and Thoracic Surgery of Nanchong Central Hospital in Sichuan Province from January 2019 to December 2022 under ultrasound or contrast-enhanced CT guidance and chest CT examination and percutaneous lung biopsy were collected into the HIS system. Patients included in the study were further identified in strict accordance with the inclusion and exclusion criteria. Before the interventional surgery, the patient had passed the hospital’s medical ethics review and signed an informed consent form for enhanced CT examination or contrast-enhanced ultrasound and percutaneous biopsy. According to different guidance methods, it is divided into contrast-enhanced ultrasound guidance group (the CEUS group) and enhanced CT guidance group (the CECT group). Among them, there were 196 cases in the CEUS group and 224 cases in the CECT group. The HIS system collects the general information of these patients (including the number of hospitalizations, name, gender, age, smoking status) and discharge diagnosis. The needle biopsy data were collected in the PACS system, including the selected guidance method, the location and size of the lesion, whether there was necrosis and liquefaction in the lesion, the number of punctured pleura, postoperative complications, and histopathological diagnosis results (**Figure 1**).

### 2.2 Inclusion criteria

1. Chest CT (including chest conventional CT and enhanced CT) examination confirmed the existence of peripheral pulmonary masses, and the sputum cytology and other bacteriological examination and other conventional tests failed to clarify the cause or obtain accurate pathological diagnosis.
2. The lesions in the CEUS group could all be clearly visualized by conventional ultrasound examination.
3. The patient ‘s imaging and pathological data and course records were detailed and complete.

### 2.3 Exclusion criteria

1. The pathological nature of lung lesions was confirmed before operation.
2. Patients who could not accept enhanced CT examination or contrast-enhanced ultrasound examination: such as pregnant or lactating patients, diabetic patients with hyperthyroidism, asthma, allergies to iodine drugs, renal insufficiency or receiving metformin treatment.
3. Patients whose lesions lesions could not be clearly visualized by ultrasound examination due to the excessively small size of the lesions or obstruction by structures such as the anterior ribs.
4. Imaging examination found hemangioma or vascular malformation.
5. Patients with conscious or mental disorders, who could not tolerate the puncture process or could not control breathing.
6. Patients with emphysema, pulmonary bullae, pulmonary heart disease, severe cardiac insufficiency and respiratory dysfunction.
7. Infection at the puncture site, or those with coagulation dysfunction or bleeding tendency.

### 2.4 Instruments and reagents

The instrument mainly includes: Mindray DC-7 produced by Mindray Company in Shenzhen, China and LOGIQ E9 color Doppler ultrasound diagnostic instrument produced by GE Company in the United States; german Siemens 64-slice spiral CT machine; 18G automatic cutting needle (Bad Magnum, Bard City, USA).

The main reagents include SonoVue ultrasound contrast agent (1.5ml), Bracco, Italy), iohexol (3.5g/L, GE), 1% lidocaine, 10% formalin, iodophor, etc.

### 2.5 Imaging-guided biopsy procedures

#### 2.5.1 Preparation before puncture

1. The puncture physician needs to confirm that the patient has no contraindications for enhanced CT examination or contrast-enhanced ultrasound examination.
2. Confirmed that the patient ‘s coagulation function and blood routine and other related examinations were not significantly abnormal. Prothrombin ratio ≥50%, international normalized ratio (INR) ≤1.6, platelet count ≥60×10^9^/L, white blood cell (WBC) count ≥3×10^9^/L were required.
3. Before the puncture, introduce the purpose of the puncture operation, the operation process, the possible complications, the matters needing attention of the patients and their families during and after the operation, and obtain the consent of the patients and their families and sign the informed consent.
4. Check the patient ‘s chest CT to determine the location and size of the lesion. According to the location of the lesion indicated by the CT examination, the lesion was found by conventional Doppler ultrasound and the lesion was clearly displayed.
5. Train the patient ‘s breathing and breath holding movements to improve the stability of the patient ‘s breathing phase during each breath holding. For those with mental stress and irritating cough, sedation or antitussive drugs can be properly controlled.

#### 2.5.2 Puncture process

**Figure 2:**
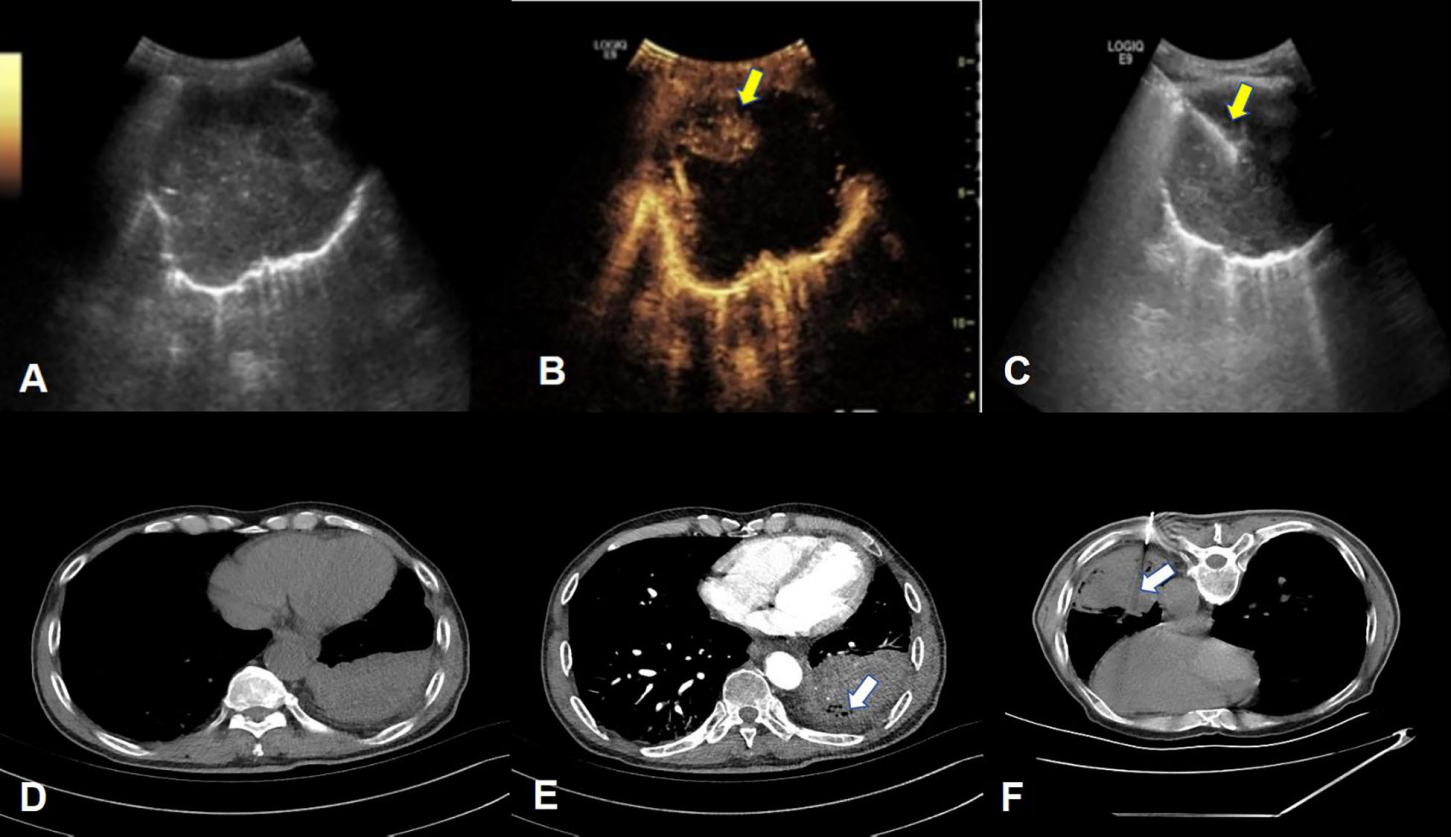
Example of percutaneous lung biopsy guided by contrast-enhanced ultrasound and contrast-enhanced CT. **(A–C)** respectively show the two-dimensional gray-scale ultrasound, contrast-enhanced ultrasound and ultrasound-guided puncture needle tract of PPLs. **(B)** clearly shows extensive liquefaction necrosis in the lesion without enhancement. The puncture needle tract shown in **(C)** avoids the non-enhancement area. (yellow arrow), the final pathological result of this patient was lung squamous cell carcinoma; **(D–F)**, respectively show the normal CT, enhanced CT and CT-guided puncture needle path of the lesion, **(E)** shows the local non-enhancing area within the lesion (white arrow), Figure The puncture needle track shown in **(F)** passed through the non-enhancing area and reached the deep tumor-enhancing area. The final pathological result of this patient was lung adenocarcinoma.

Contrast-enhanced ultrasound-guided percutaneous lung biopsy A 3.5~5.0MHz convex array probe was used to examine the location, shape, size, internal echo, blood supply characteristics, and relationship with the chest wall and surrounding tissues. After the conventional ultrasound examination was completed, the contrast mode was switched. The assistant was instructed to quickly absorb the 2.4ml contrast agent suspension through the elbow vein, and then flush the tube with 5ml 0.9% sodium chloride solution. The lesion and surrounding tissue were continuously observed for at least 3 minutes, continuously and dynamically observe the perfusion of the lesion and surrounding tissue, determine the internal structure and properties of the lesion, and locate the active tumor area (**Figures 2A–C**). The puncture site is disinfected, draped, and local anesthetized, and real-time ultrasound-guided lung biopsy is performed on the puncture target. General observations were made on the obtained tissue strips. If the material was not qualified, it was necessary to adjust the depth and angle of the needle and repeat the puncture. The punctured tissue was fixed with 10% formalin solution and immediately sent for pathological examination. The number of pleural punctures and tissue specimens were recorded in detail.

Enhanced CT-guided percutaneous lung biopsy: Select the appropriate position, select the best puncture level and puncture target under CT guidance, and place the metal positioning mark at the proposed puncture point. The proposed puncture point is routinely disinfected and toweled. 2% lidocaine infiltration anesthesia until the visceral pleura, place the puncture needle under the skin of the puncture point, and once again CT scan confirms the direction and depth of the needle. Immediately ask the patient to hold his breath, and the puncture needle enters the lesion at the established angle and depth. After scanning again to confirm that the puncture needle reaches the target position of the mass, start the biopsy gun for biopsy, and observe whether the tissue strip meets the standard (**Figures 2D–F**). If necessary, repeat the above operation process. The biopsy specimens were fixed with 10 formalin solution and sent for pathological examination. After the puncture was completed, the needle insertion site, path, depth and number of punctures were recorded.

#### 2.5.3 Complications after puncture

After the biopsy procedure is completed, the patient is examined and asked about any discomfort, and an ultrasound or chest X-ray is performed to determine whether pneumothorax and bleeding have occurred. A small amount of pneumothorax does not require special treatment and can be spontaneously absorbed. In such cases, patients are instructed to undergo close observation. When a massive pneumothorax occurs (more than 30% of the lung tissue is compressed), closed chest drainage may be employed. If major bleeding occurs, hemostatic drugs should be administered to the patient via injection as expeditiously as possible, or surgical treatment should be promptly implemented. If there is no obvious discomfort after puncture, the patient should return to the ward to rest in bed. Vital signs, including blood pressure, respiratory rate, pulse and other indicators should be closely observed within 6 hours after puncture, and the patients with discomfort symptoms should be immediately arranged to undergo X - ray chest radiography to detect the presence of pneumothorax at an early stage.

#### 2.5.4 Evaluation criteria

**Figure 3:**
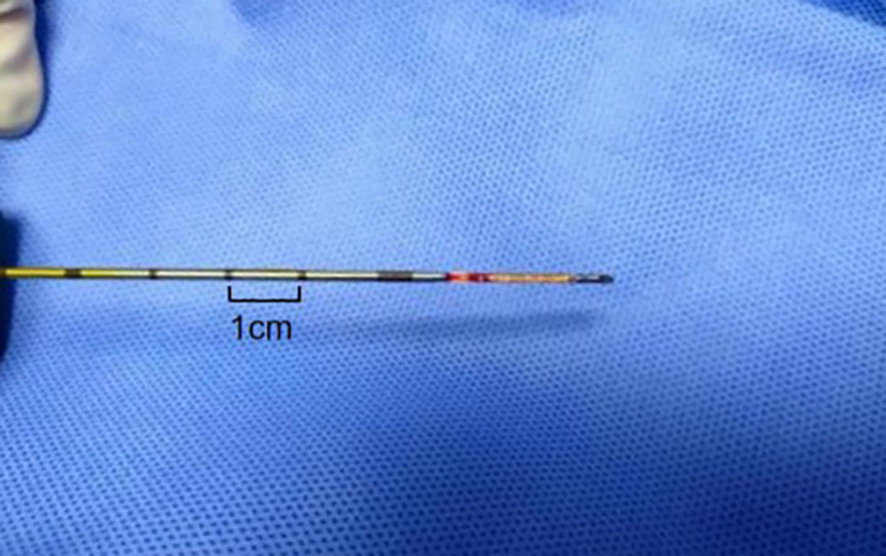
Percutaneous lung biopsy tissue strip. The tissue strip is a light red complete tissue strip with a length >1cm.

(1) Satisfactory sampling: The length of the specimen obtained by biopsy is not less than 1cm of fish-like or light red complete tissue strips, and there is sufficient amount of cells for histopathological examination (**Figure 3**). If the specimen received by the Department of Pathology is necrotic or mucus-like tissue, or suggests that the puncture tissue has less cells or punctures into muscle tissue, alveolar tissue or necrotic tissue without clear diagnostic significance, the sampling is not satisfactory.
(2) Accurate diagnosis: Malignant tumor cells or benign cells with clear diagnostic significance can be accurately found in the puncture specimen, and the puncture result is consistent with the final clinical diagnosis as the standard for accurate puncture. Malignant lesions refer to the presence of obvious malignant tumor cells in the lesions as indicated by puncture histopathology or confirmed malignant by postoperative pathology; benign lesions refer to benign cells with clear diagnostic significance found in the puncture specimen tissue and confirmed by surgery. Or patients without surgery were followed up for half a year or more after treatment. Imaging examination shows that the lesion disappears or shrinks, and it can be diagnosed as benign. Otherwise, the pathological diagnosis is considered unclear.
(3) First puncture diagnostic accuracy: the probability of obtaining accurate pathological results from the first percutaneous biopsy (statistical diagnostic accuracy is the first puncture result, excluding subsequent repeated biopsies).
(4) Postoperative complications: ① Pneumothorax: The patient develops chest pain and dyspnea during or after puncture. Chest X-ray or CT scan shows air in the chest cavity on the puncture side and compression of lung tissue. ② Bleeding: Hemoptysis or blood in sputum occurs during or after puncture. CT scan shows ground glass opacities or blunt costophrenic angles around the needle path. An ultrasound scan shows a new dark area of fluid in the costophrenic angle suggesting thoracic or pulmonary hemorrhage.
(5) Puncture time: the time from the patient entering the examination room to the completion of puncture.

#### 2.5.5 Observation indicators

(1) The satisfaction rate of the first puncture sampling.
(2) Accuracy of first puncture diagnosis.
(3) Puncture pleural times, puncture time.
(4) The first puncture diagnostic accuracy of benign and malignant lesions.
(5) Incidence of postoperative complications (pneumothorax, hemorrhage).

### 2.6 Statistical analysis

The above data were analyzed by SPSS 21.0 statistical software (SPSS Inc., Chicago, Illinois, USA), and the enumeration data were expressed by rate (%). The comparison of the two guiding methods was performed by random design χ² test or Fisher exact probability method. Measurement data conforming to normal distribution and homogeneity of variance were expressed as mean ± standard deviation, and two independent sample *t*-test was used. The measurement data that do not conform to the normal distribution and homogeneity of variance are expressed by the median (quartile interval) [M(P_25_, P_75_)], and the nonparametric Mann-Whitney U test, two-sided test, test level *α* = 0.05, with *P* < 0.05 for the difference was statistically significant.

## 3 Results

### 3.1 Patient characteristics

A total of 420 patients were included in this study. There were 196 cases in the CEUS group, including 152 males and 44 females. The mean age was (66.7 ± 10.7) years (range, 19-89 years). The lesion location: 107 cases of right lung, 89 cases of left lung; there were 123 smokers and 73 non-smokers. The maximum diameter of the lesion ranged from 1.5 to 12.3cm, with an average of (5.9 ± 2.5) cm. Conventional ultrasound detected 55 cases of liquefaction necrosis in the lesion, and contrast-enhanced ultrasound detected 106 cases of liquefaction necrosis. There were 224 patients in the CECT group, including 161 males and 63 females, aged 24-86 years, with an average age of (64.8 ± 10.1) years. The lesion location: 115 cases of right lung, 109 cases of left lung; there were 144 smokers and 80 non-smokers. The maximum diameter of the lesion ranged from 1.3 to 12.4cm, with an average of (4.5 ± 2.2) cm. In the CECT group, 90 cases of liquefaction necrosis were detected. There was no significant difference in age, sex, location and smoking between the two groups (*P* > 0.05). There was significant difference in the maximum diameter of the lesion and the detection of liquefaction necrosis between the two groups (*P* < 0.05) (**Table 1**).

**Table 1:** Patient demographics and lesion characteristics.

Characteristics	CEUS	CECT	*χ^2^*/t	P
Age (years)	66.7 ± 10.7	64.8 ± 10.1	1.856	0.0674
Male, n (%)	152 (77.6)	161 (71.9)	1.774	0.217
Lesion location				
Right lung, n (%)	107 (54.6)	115 (51.3)	0.092	0.764
Smoking, n (%)	122 (62.2)	144 (64.3)	0.106	0.761
Lesion size (cm)	5.9 ± 2.5	4.5 ± 2.2	5.955	<0.001
Necrosis, n (%)	106 (54.1)	79 (35.3)	4.931	<0.029

Data are expressed as mean ± standard deviation if not otherwise specified.

### 3.2 Comparison of the average number of pleural punctures and time-consuming between the two groups

The number of pleural punctures in the CEUS group was about 1 ~ 5 times, with an average of 2.4 times. The puncture time was about 12 ~ 42 minutes, with an average time of about 23.6 minutes per patient. The number of pleural punctures in CECT group was about 1-11 times, with an average of 4.1 times; the puncture time was about 30 ~ 62 minutes, with an average time of about 42.4 minutes per patient. The number of pleural punctures in the CEUS group was less than that in the CECT group, and the puncture time was shorter. The difference was statistically significant (*P* < 0.05) (**Table 2**).

**Table 2:** Comparison of two groups of puncture.

	CEUS	CECT	χ²/t	P
Number of punctures	2.4 ± 0.9	4.1 ± 1.4	15.330	<0.001
Puncture time (min)	23.6 ± 4.9	42.4 ± 7.4	31.219	<0.001

### 3.3 The satisfaction rate and diagnostic accuracy of the first puncture were compared between the two groups.

In CEUS group, 183 cases obtained satisfactory tissue specimens, and 13 cases were unsatisfactory. In the CECT group, 202 cases obtained satisfactory tissue specimens, and 22 cases were unsatisfactory. The overall satisfaction rate of the first puncture in CEUS group was about 93.4%, and the overall satisfaction rate of the CECT group was about 90.2%. The difference was not statistically significant (*P* > 0.05). Among them, in the lesion diameter 3-6cm group, the satisfaction rate of the CEUS group was 98.6%, which was higher than that of the CECT group (89.6%), and the difference was statistically significant (*P* < 0.05). In the lesion diameter < 3cm group, the satisfaction rate of the CEUS group was about 71.0%, which was lower than that of the CECT group (88.3%), and the difference was statistically significant (*P* < 0.05). In the lesion diameter > 6cm group, the satisfaction rate of the CEUS group was 96.8%, and the satisfaction rate of the CECT group was 93.9%. The difference was not statistically significant (*P* > 0.05). In the CEUS group, 170 cases were diagnosed accurately and 26 cases were diagnosed incorrectly. In the CECT group, 192 cases were diagnosed accurately and 32 cases were diagnosed incorrectly. The overall diagnostic accuracy of the first puncture between the two groups was about 86.7% and 85.7%, respectively, and the difference was not statistically significant. In the lesion diameter < 3cm group, the diagnostic accuracy of the CEUS group was 64.5%, which was lower than 86.7% of the CECT group, and the difference was statistically significant (*P* < 0.05). In the lesion diameter 3-6cm group, the diagnostic accuracy of the CEUS was 95.8%, which was higher than that of the CECT group (85.2%), and the difference was statistically significant (*P* < 0.05). The diagnostic accuracy of the first puncture in the CEUS group and the CECT group in the lesion diameter > 6 cm group was about 87.1% and 85.7%, respectively, and the difference was not statistically significant (*P* > 0.05) (**Table 3**).

**Table 3:** Comparison of the satisfaction rate and diagnostic accuracy of the first puncture in the two groups.

	CEUS	CECT	χ²/t	P
Overall material satisfaction rate	183/196	202/224	1.391	0.289
Lesions < 3cm	22/31	53/60	4.254	0.047
Lesions 3~6 cm	71/72	103/115	5.601	0.018
Lesions>6cm	90/93	46/49	0.142	0.415
Overall diagnostic accuracy	170/196	192/224	0.091	0.779
Lesions < 3cm	20/31	52/60	6.071	0.027
Lesions 3~6 cm	69/72	98/115	5.224	0.027
Lesions>6cm	81/93	42/49	0.053	0.801

### 3.4 The first puncture diagnosis of benign and malignant diseases was compared between the two groups

In CEUS group, 141 cases of lung malignant tumors and 55 cases of benign lesions were finally diagnosed. There were 56 cases of adenocarcinoma, 56 cases of squamous cell carcinoma, 17 cases of neuroendocrine tumors, 6 cases of metastatic tumors and 6 cases of other malignant tumors. There were 36 cases of inflammatory lesions, 12 cases of tuberculosis and 7 cases of other benign lesions. In the CECT group, 183 cases of lung malignant tumors and 41 cases of benign lesions were finally diagnosed. There were 86 cases of adenocarcinoma, 51 cases of squamous cell carcinoma, 24 cases of neuroendocrine tumors, 12 cases of metastatic tumors and 10 cases of other malignant tumors. There were 28 cases of inflammatory lesions, 11 cases of tuberculosis and 2 cases of other benign lesions. There was no significant difference in the nature and pathological types of lesions between the two groups (*P* > 0.05).

In the CEUS group, the number of cases with satisfactory first-puncture sampling was 183, while the number of cases with unsatisfactory sampling was 13. Among the 183 cases with satisfactory sampling, 121 cases were diagnosed with pulmonary malignant tumors, and the remaining 62 cases were initially indicated as non- malignant lesions. During the follow - up, it was discovered that among these 62 cases, 13 cases had incorrect initial diagnoses, which were eventually diagnosed with pulmonary malignant tumors through repeated puncture or postoperative pathological examination. Among the 13 cases with unsatisfactory first-puncture sampling, 7 cases were confirmed as pulmonary malignant tumors by repeated puncture or surgical pathological results. The postoperative pathological results of 2 cases showed inflammatory pseudotumors, and 4 cases were determined to be inflammatory nodules based on the follow-up. (**Table 4**).

**Table 4:** The results of percutaneous lung biopsy guided by CEUS and final clinical diagnosis in 196 cases.

The first puncture pathological diagnosis	Clinical diagnosis		Total
	Malignant	Benign	
Malignant	121	0	121
Benign	13	49	62
Uncertain	7	6	13
Total	141	55	196

In the CECT group, there were 202 cases with satisfactory first-time puncture sampling and 22 cases with unsatisfactory first-time puncture sampling. Among the cases with satisfactory first-time puncture sampling, 160 cases were diagnosed with pulmonary malignant tumors, and 42 cases were indicated as non-malignant lesions, among which it was found that there were 8 misdiagnosed cases during follow-up. After repeated punctures or postoperative pathological examinations, these 8 cases were confirmed to have pulmonary malignant tumors. Among the cases with unsatisfactory first - time puncture sampling, 15 patients underwent repeated enhanced CT - guided percutaneous lung biopsies. The results showed that 9 cases were pulmonary malignant tumors, 2 cases were pulmonary tuberculosis, and 4 cases were inflammatory lesions. The other 7 patients received surgical treatments, and the postoperative pathological results revealed that 6 cases were pulmonary malignant tumors and 1 case was a fungal nodule (**Table 5**).

**Table 5:** The results of percutaneous lung biopsy guided by CECT and final clinical diagnosis in 224 cases.

The first puncture pathological diagnosis	Clinical diagnosis		Total
	Malignant	Benign	
Malignant	160	0	160
Benign	8	34	42
Uncertain	15	7	22
Total	183	41	224

The diagnostic accuracy of the first puncture of percutaneous lung biopsy guided by CEUS group and CECT group for the diagnosis of lung malignant tumors was 85.8% and 87.4%, respectively. Meanwhile, the diagnostic accuracy of the first puncture for benign lesions was approximately 89.1% and 82.9%, respectively. There was no significant difference in the first time diagnostic accuracy for both lung malignant tumors and lung benign lesions between the two groups (*P* > 0.05) (**Table 6**).

**Table 6:** Comparison of diagnostic accuracy of benign and malignant lesions in the first puncture between the two groups.

	CEUS	CECT	χ²/t	P
The first diagnostic accuracy of malignant tumor	121/141	160/183	0.181	0.742
The first diagnostic accuracy of benign lesions	49/55	34/41	0.762	0.548

### 3.5 The incidence of pneumothorax and bleeding after puncture was compared between the two groups

In the CEUS group, 6 case had pneumothorax after puncture and 3 cases had hemorrhage after puncture. In the CECT group, there were 18 cases of pneumothorax and 7 cases of bleeding after puncture. The proportion of pneumothorax after puncture in the CEUS group was lower, and the difference was statistically significant (*P* < 0.05) (**Table 7**).

**Table 7:** Comparison of the incidence of pneumothorax and hemorrhage between the two groups.

	CEUS	CECT	*χ^2^*/t	P
Pneumothorax	6/196	18/224	4.801	0.034
Hemorrhage	3/193	7/224	1.143	0.349

## 4 Discussion

This article evaluates the differences between the two guidance methods by comparing the number of pleural punctures, puncture time, first puncture satisfaction, diagnostic accuracy, and complication rate. Compared with the CECT group, the CEUS group required fewer pleural punctures on average (2.4 ± 0.9 times vs. 4.1 ± 1.4 times) and shorter puncture time (23.6 ± 4.9 minutes vs. 42.4 ± 7.4 minutes), with a statistically significant difference(*P*<0.001). Previous studies have shown that, compared with the time consuming and the number of pleural punctures of conventional CT-guided percutaneous lung biopsy, ultrasound-guided percutaneous lung biopsy requires less time and fewer punctures (16–18). The results of our study show that both CEUS-guided and enhanced CT-guided percutaneous lung biopsy can achieve high diagnostic accuracy. Nevertheless, the satisfaction rate and diagnostic accuracy of the first puncture are related to the size of the lesion. When the diameter of the lesion is <3 cm, the specimen satisfaction and diagnostic accuracy of the CEUS group are lower than those of the CECT-group (71.0% vs. 88.3%, 64.5% vs. 86.7%). When the diameter of the lesion ranges from 3 to 6 cm, the specimen satisfaction and diagnostic accuracy of the CEUS group were higher than those of the CECT group (98.6% vs. 89.6%, 95.8% vs. 85.2%), and the above differences were statistically significant. However, when the diameter of the lesion was >6cm, there was no significant difference in specimen satisfaction rate and diagnostic accuracy between the two methods. In terms of complications, the incidence of pneumothorax (3.1% vs. 8%) was lower in the CEUS group, while the incidence of bleeding (1.5% vs. 3.1%) was not significantly different between the two groups.

Ultrasound is not commonly used for routine examination of lung tumors due to the significant difference in acoustic impedance between lung tissue and air, which leads to strong reflection of ultrasound waves. Nevertheless, peripheral lung cancer is typically located at the edge of lung tissue, close to the chest wall. In such cases, ultrasound can clearly display the boundary between the tumor and the surrounding tissue, facilitating the determination of the tumor’s location and size. Ultrasound - guided biopsy can then be performed to confirm the pathological type of the tumor. The ultrasound - guided puncture operation is relatively easy to position, and there is no need for frequent confirmation or adjustment of the puncture point. During the puncture process, real - time monitoring of needle insertion is possible, allowing for timely adjustment of the needle’s depth and angle to optimize the puncture process’s continuity, which can significantly reduce the number of pleural punctures and shorten the puncture time. Yamamoto (2) also believed that ultrasound-guided percutaneous lung biopsy was more effective than CT-guided biopsy in the diagnosis of large mass lesions. In contrast, CECT-guided percutaneous lung biopsy often uses a step-by-step puncture technique, which requires multiple CT scans to ensure that the needle tip reaches the target position accurately. This process increases the frequency of the needle penetrating the pleura and prolongs the needle’s dwell time within the lungs. Additionally, due to the time-consuming nature of the CT-guided puncture operation, patients may have difficulty maintaining consistent breathing cooperation, which may cause the location of the lesion to move, thereby affecting the accuracy of puncture. This requires doctors to adjust the angle or depth of the puncture needle multiple times, further increasing the number of pleural punctures and overall procedure time (19).

CT scanning can obtain high-quality reconstructed images in a short time. It can also monitor and adjust the specific position and direction of the puncture needle at any time. It can accurately locate the puncture point, needle insertion angle and needle insertion depth, and can observe it from various angles (20). By precisely defining the positional relationship between the puncture needle and the lesion, it ensures that the puncture needle reaches the intended puncture site. Immediately after the puncture, a scan can be performed to detect the occurrence of any complications. Especially for small lesions, small angle deviations and the patient’s breathing pattern can affect the puncture path. These factors may prevent the needle tip from accurately reaching the puncture target, ultimately leading to puncture failure. When the puncture needle penetrates the atelectatic lung tissue around the lesion, it is easy to cause a large number of lung epithelial tissue cells or blood cells to appear in the sample, resulting in insufficient cells of diagnostic significance and affecting the diagnosis due to unqualified sampling. Enhanced CT examination is not only not affected by gas and bone occlusion, but also has high resolution of thin-section scanning images, and can also display lesions smaller than 1cm. Therefore, our study shows that for pulmonary nodules smaller than 3 cm, compared with CEUS guidance, enhanced CT-guided percutaneous lung biopsy exhibits a higher satisfaction rate of the first - time sampling and diagnostic accuracy.

However, within the lesions, there may exist local areas with abundant blood supply or liquefied necrosis areas. During actual operations, puncturing these areas may elevate the probability of bleeding or result in ineffective punctures. In this study, when the lesion size ranged from 3 ~ 6cm, the satisfaction and diagnostic accuracy of the CEUS group were higher than those of the CECT group. The reason may be that as the lesions increase in size, liquefactive necrosis is more prone to occur within the lesions. The liquefaction areas vary in size and shape. CEUS, being a real-time dynamic imaging, can observe in real-time across multi-section, and can accurately distinguish the main blood vessels inside the lesion and the smaller non - enhancing liquefaction necrosis areas, so as to better guide the puncture (19). In contrast, for enhanced CT, the enhancement process may be influenced by factors such as the scanning phase, the thickness of the scanning layer and its influence on the tomographic image. It is less satisfactory in displaying the smaller liquefied necrotic areas inside the lesion and cannot monitor the puncture process in real - time. Needle insertion in this case can easily lead to sampling failure and a reduction in diagnostic accuracy. There is high-level evidence that rapid on-site evaluation (ROSE) before CT-guided puncture can help improve diagnostic accuracy without increasing the incidence of complications (20). Nevertheless, this method conducts a quick evaluation of the needle route and puncture target area based on the existing enhanced CT results before puncture, lacking the ability to provide real-time guidance. Simultaneously, on - site puncture evaluation also prolongs the overall operation time. When the diameter of the lesion is greater than 6cm, both CEUS and CECT can effectively distinguish between liquefied necrotic area and atelectasis of lung tissue. In addition, for the mass with a larger diameter, it is farther from the pleura, allowing for more flexible selection of the puncture point and puncture angle during the puncture process. At this time, the visual monitoring under the guidance of CEUS for the active area around the puncture is of particularly important. The CECT group can be re-evaluated through the ROSE protocol to determine the best puncture path. In this study, when the diameter of the lesion was >6cm, both groups selected the puncture target area through enhanced imaging of the lesion. Consequently, the satisfaction rate and diagnostic accuracy of the first puncture in the large lesion (>6 cm) group were similar between the CEUS group and the CECT group, and the difference was not statistically significant.

Although percutaneous transthoracic lung biopsy is a minimally invasive method, it is still an invasive examination, which may give rise to various complications, including pneumothorax, hemorrhage, subcutaneous and mediastinal emphysema, infection, tumor needle implantation, pleural reaction and other complications. Among them, pneumothorax and bleeding after puncture are the most common complications. Previous research has indicated that the incidence of pneumothorax and bleeding in CT-guided percutaneous lung biopsy was 10 ~ 30% and 1 ~ 47% respectively (20). Pneumothorax is caused by the injury of lung and visceral pleura during biopsy. There are many factors affecting the occurrence of pneumothorax. For instance, the diameter of the puncture needle will affect the occurrence of pneumothorax. Studies have shown that the incidence of pneumothorax in the 16-needle group is significantly higher than that in the 18-needle group (6). Additionally, the size of the lesion and the length of the pleural contact also have an important impact on the occurrence of pneumothorax. When the lesion is less than 1cm or the length of the pleural contact is less than 1cm, the difficulty of puncture increases, this often leads to the puncture needle traversing beyond the edge of the lesion and damaging the surrounding lung tissue, thereby resulting in pneumothorax (21). Hemorrhage is another common complication of percutaneous transthoracic lung biopsy. There are many factors affecting the occurrence of hemorrhage. The location and size of the lesion, the gauge of the puncture needle, the number of punctures attempts, the puncture position, the depth of puncture, the pulmonary artery pressure, coagulation function, as well as the nature of the tumor and its blood supply, all contribute to the likelihood of hemorrhage (15). Through multivariate Logistic regression analysis, Fu et al. (19) found that lesions ≤3cm, supine puncture, puncture times ≥2 times, puncture depth ≥4cm were more likely to bleed. The smaller the lesion, the more difficult the positioning, the more times the puncture needle needs to be adjusted, the greater the depth of the puncture, the greater the damage to the lung tissue. In addition, the patient ‘s position is closely related to complications such as pneumothorax and bleeding (2, 22–24). The stability of the patients in the lateral position was lower than that in the supine position and the prone position. In addition, when the patient is in the lateral position, the pulmonary mobility on the puncture side is augmented, which may also increase the risk of lung parenchyma injury. In this study, a total of 6 cases of small pneumothorax (3.1%) occurred in the CEUS group, which was close to the research of other scholars (19). Among them, 3 cases occurred in the small lesion group. Due to the small lesion, the lung tissue was damaged at the first puncture, the gas overflowed, and the ultrasonic exploration was blocked, resulting in the failure of sampling. There were a total of 18 cases of pneumothorax (8.0%) in the CECT group, of which 2 cases underwent closed thoracic drainage due to the large amount of pneumothorax. The incidence of pneumothorax after puncture in the CECT group was higher than that in the CEUS group. The difference was statistically significant, which was consistent with the previous relevant literature reports. Upon analysis, the possible reason is that during the entire process of ultrasound - guided pleural puncture, the number of pleural punctures is relatively low, and the procedure time is very short. The whole process of biopsy can be observed in real time, and it can cooperate with the patient ‘s respiratory movement, and insert the needle immediately after the patient holds their breath, so as to avoid damage to normal lung tissue. CEUS and CECT can observe the lesion and the blood vessels around the puncture path, so the whole process of puncture can avoid the puncture to the thick blood vessels and reduce the occurrence of bleeding. In this study, the incidence of bleeding after puncture was low. There were 3 cases in the CEUS group and 7 cases in the CECT group. All of them showed slight bleeding. The patients were instructed to rest in bed without special intervention.

The current study has several limitations. First, this is a single-center retrospective study with a small sample size and unintentional selection bias in statistics. Second, there is a certain degree of selection bias in the selection of cases. The choice of biopsy method for PPLs is decided by the clinician who formulates the plan based on preoperative imaging results and personal preferences. Currently, there are no established standards to guide the selection of methods. This introduces selection bias and reduces general applicability. Third, postoperative complication detection in the CEUS group was assessed by ultrasound and chest radiography, and the incidence of complications may be underestimated compared with postoperative CT scan assessment in the CECT group. Fourth, our study did not include patients with emphysema, Patients with emphysema who underwent ultrasound examination may have poor imaging quality due to the large acoustic impedance difference between lung tissue and air, so these patients were excluded from the collection of cases. This is also the limitation of ultrasound examination, and the relevant content has been supplemented in the article. Fifth, the ability of ultrasound to allow a tissue sampling sufficient to allow a conclusive mutational analysis of the tumor is not yet know. In addition, although this study counted the puncture time required for the two guidance methods, it did not count the economic costs of the two methods.

## 5 Conclusions

CEUS is superior to CECT in reducing the number of punctures, shortening puncture time, and reducing the incidence of pneumothorax, and is especially suitable for the diagnosis of medium-sized lesions. However, for lesions less than 3 cm in diameter, CECT demonstrated higher specimen satisfaction and diagnostic accuracy. This suggests that diagnostic performance can be optimized by selecting appropriate guidance techniques based on lesion size and risk of complications.

## Data Availability

The datasets presented in this article are not readily available because If necessary, the author can request access to the original data. Requests to access the datasets should be directed to liujiansh@126.com.
